# Exposure to secondhand tobacco smoke is associated with reduced muscle strength in US adults

**DOI:** 10.18632/aging.102594

**Published:** 2019-12-09

**Authors:** Monica Carrasco-Rios, Rosario Ortolá, Fernando Rodríguez-Artalejo, Esther García-Esquinas

**Affiliations:** 1 Department of Preventive Medicine and Public Health, School of Medicine, Universidad Autónoma de Madrid/ IdiPAZ, Madrid, Spain; 2 CIBER of Epidemiology and Public Health (CIBERESP), Madrid, Spain

**Keywords:** secondhand tobacco smoke, muscle strength, cotinine, musculoskeletal disease

## Abstract

Secondhand tobacco smoke (SHS) exposure is a well-established risk factor for several diseases in adults. Despite the evidence that active tobacco smoke is harmful for the muscles, the association between SHS and muscle strength is still uncertain.We analyzed data from 5390 nonsmoking U.S. adults aged >30 years who participated in the National Health and Nutrition Examination Survey (NHANES) 2011-2014. Exposure to SHS was assessed with serum cotinine concentrations. Grip strength was measured using a Takei digital handgrip dynamometer, and combined grip strength was calculated as the sum of the largest reading from each hand. Median (interquartile range) serum cotinine and grip strength were 0.015 ng/mL (IQR 0.011-0.36) and 65.5 kg (IQR 53.4-86.4), respectively. After adjusting for sociodemographic, anthropometric, health-related behavioral, and clinical risk factors, the highest (0.047-9.9 ng/mL) vs lowest (≤0.011 ng/mL) quartile of serum cotinine was associated with a reduction in combined grip strength of 1.41 kg (95%CI: -2.58, -0.24), p-trend=0.02. These results were consistent across socio-demographic and clinical subgroups. In the US nonsmoking adult population, even low levels of exposure to passive smoking were associated with decreased grip strength. Despite great achievements in tobacco control, extending public health interventions to reduce SHS exposure is still needed.

## INTRODUCTION

Exposure to secondhand tobacco smoke (SHS) is a global health problem [1]. In 2016, an estimated 33% of non-smoking women and 20% of non-smoking men were exposed to SHS worldwide [1]. SHS exposure in adult life increases the risk of coronary heart disease, stroke and lung cancer, with suggestive evidence that it may also be a risk factor of chronic obstructive pulmonary disease, asthma, impaired lung function, atherosclerosis, and cancers of the paranasal sinus, pharynx, larynx and breast [2, 3]. Only in 2016, SHS caused around 884,000 deaths worldwide [1].

Muscle strength is an important determinant of physical function, and is a good predictor of major health outcomes such as hospitalization [4], disability [5], and all-cause death [6]. Furthermore, there is some evidence suggesting that low muscle strength may increase the risk of cardiovascular disease, fractures and cognitive function decline [7, 8]. These deleterious health consequences of muscle strength declines make the identification of potential modifiable risk factors an important public health priority. Hence, for example, several epidemiological studies have studied the effects of poor nutritional status [9–11], excess body weight [12–14], physically strenuous work [12], or decreased leisure time physical activity [15] on muscle strength declines. However, and despite the evidence linking active tobacco smoke with reduced muscle strength [12, 16], increased risk of frailty [17] or physical disability [18], there are a very few studies that have addressed the potential effects of SHS on physical function or muscle strength declines, and none of these have included young adults [19, 20].

We hypothesized that SHS exposure is inversely associated with muscle strength both in middle-aged and older adults. To assess this hypothesis, we examined the association between SHS and grip strength in nonsmoking adults aged >30 years who participated in the U.S. National Health and Nutrition Examination Survey (NHANES) in 2011–2014.

## RESULTS

Table 1 shows the distribution of the study population by quartiles of serum cotinine. A high proportion of subjects (48.3%) had serum cotinine values below the LOD (≤0.011 ng/mL). When compared to lower quartiles of serum cotinine, the proportion of individuals in the highest quartile (0.047-9.9 ng/mL) was higher for younger men, individuals with lower education, non-Hispanic blacks, ex-smokers, ex-drinkers, and those with poor dietary quality, obesity or cardiovascular disease.

Median (IQR) concentrations of serum cotinine and combined grip strength were 0.015 ng/mL (0.011-0.36) and 65.5 Kg (53.4-86.4), respectively. Women, individuals aged > 60 years, with lower educational level, lower BMI, never drinkers, and cardiovascular or musculoskeletal disease, showed the lowest strength (Tables 1 and 2).

**Table 1:** Number of participants (weighted percentage) across quartiles of serum cotinine concentrations, overall and stratified by participants sociodemographic and clinical characteristics.

	**Serum cotinine concentration (ng/mL)**				
	**Q1 ≤0.011**	**Q2 0.015-0.02**	**Q3 0.021-0.047**	**Q4 0.047-9.9**	**p val^†^**
**Total**	2287 (48.3)	626 (11.9)	1193 (20.1)	1284(19.7)	
**Sex**					
Men	974 (44.1)	292 (47.1)	545 (45.6)	643 (49.7)	
Women	1313(55.9)	334 (52.9)	648 (54.4)	641 (50.3)	<0.001
**Age, years**					
30-40	425 (19.0)	127 (20.6)	239 (21.0)	316 (25.6)	
41-50	470 (22.6)	128 (22.7)	249 (22.8)	255 (21.5)	
51-60	418 (21.0)	139 (26.2)	256 (24.8)	259 (24.5)	
>60	974 (37.4)	232 (30.5)	449 (31.4)	454 (28.4)	<0.001
**Educational level,**					
<High School	377 (10.0)	107 (10.2)	259 (13.9)	346 (19.0)	
High School	383 (15.3)	114 (15.6)	238 (19.5)	330 (27.1)	
> High School	1527 (74.7)	405 (74.2)	696 (66.6)	608 (53.9)	<0.001
**Race/Ethnicity**					
Non-Hispanic white	1132 (76.9)	232 (67.8)	418 (65.2)	443 (60.5)	
Non-Hispanic black	292 (5.0)	106 (7.5)	298 (12.6)	420 (17.6)	
Mexican-American	320 (7.4)	97 (10.0)	122 (7.5)	129 (7.9)	
Other	543 (10.7)	191 (14.7)	355 (14.7)	292 (14.0)	<0.001
**Country of birth**					
US	1550 (83.1)	382 (79.7)	713 (76.3)	939 (82.9)	
Other	737 (16.9)	244 (20.3)	480 (23.7)	345 (17.1)	<0.001
**BMI**					
Under-/ normoweight	640 (28.4)	177 (25.0)	324 (24.2)	260 (17.5)	
Overweight	807 (37.0)	205 (36.7)	417 (36.6)	407 (33.3)	
Obese	840 (34.6)	244 (38.3)	452 (39.3)	617 (49.2)	<0.001
**Physical activity (METs-hour/week)^††^**					
1^st^ tertile	854 (33.3)	239 (37.9)	453 (34.2)	528 (36.9)	
2^nd^ tertile	776 (35.6)	214 (32.2)	382 (33.1)	367 (29.4)	
3^rd^ tertile	657 (31.1)	173 (29.9)	358 (32.7)	389 (33.7)	0.047
**Smoking, n (% weighted)**					
Never	1647 (71.8)	449 (72.1)	842 (66.1)	792 (60.8)	
Ex-smoker	640 (28.2)	177 (27.9)	351 (33.9)	492 (39.2)	<0.001
**Alcohol consumption, n**					
Never	403 (13.7)	97 (8.6)	195 (12.6)	195 (13.3)	
Ex-drinker	483 (18.4)	152 (21.9)	255 (20.4)	330 (24.4)	
Current	981 (53.3)	272 (54.7)	473 (51.2)	512 (47.3)	
Unknow	420 (14.6)	105 (14.8)	270 (15.8)	247 (15.0)	<0.001
**Diet quality, n**					
Excellent/very good	852 (42.6)	214 (35.1)	389 (34.7)	382 (29)	
Good	966 (40.6)	266 (43.5)	539 (45.9)	551 (41.7)	
Fair/poor	469 (16.8)	146 (21.4)	265 (19.4)	351 (29.3)	<0.001
**Diagnosed disease, n**					
Cardiovascular disease	250 (8.7)	64 (9.2)	120 (8.5)	173 (12.5)	0.030
Respiratory disease	342 (15.6)	98 (16.0)	185 (15.4)	231 (19.6)	0.117
Musculoskeletal disease	673 (28.5)	175 (28.5)	330 (28.3)	414 (31.4)	0.063
Cancer	310 (14.3)	69 (16.0)	109 (11.6)	109 (9.3)	<0.01
Hypertension	984 (38.8)	256 (37.1)	522 (39.5)	585 (43.5)	0.242
Diabetes	395 (12.5)	110 (15.2)	226 (14.6)	263 (17.0)	0.104
**Number of drug treatments**					
None	860 (38.0)	219 (33.9)	442 (37.3)	466 (37.2)	
1-2	286 (13.7)	98 (16.3)	140 (12.7)	163 (11.6)	
>2	1141 (48.3)	309 (49.8)	611 (50.0)	655 (51.2)	0.341

Data are based on 5,390 adults aged >30 years from the US nonsmoking general population.

^**†**^ P values are based on the chi-square test.

^**††**^ Cutoff values (men/women) were ≤12/≤4 (tertile 1), 12 to ≤48 /4.1 to ≤28 (tertile 2), and >48/28. (tertile 3) METs-hour/week.

**Table 2:** Median (interquartile range [IQR]) serum cotinine and muscle strength in adults aged >30 from the US nonsmoking general population by participants sociodemographic and clinical characteristics.

**Characteristics**	**n (weighted %)**	**Serum cotinine (ng/mL)**	**Combined grip strength (Kg)**
**Total**	5390 (100)	0.015 (0.011-0.360)	65.5 (53.4-86.4)
**Sex**			
Men	2454 (45.9)	0.016 (0.011-0.040)	88.5 (76.5-99.6)
Women	2936 (54.1)	0.015 (0.011-0.033)	55.1 (47.6-62.4)
**Age, years**			
31-40	1107 (20.9)	0.017 (0.011-0.046)	72.9 (60.3-95.0)
41-50	1102 (22.4)	0.015 (0.011-0.035)	72.2 (59.4-93.4)
51-60	1072 (23.1)	0.018 (0.011-0.038)	66.8 (54.4-88.9)
>60	2109 (33.6)	0.011 (0.011-0.029)	56.2 (45.6-73.5)
**Educational level**			
< High School	1089 (12.6)	0.023 (0.011-0.060)	59.0 (46.8-77.1)
High School	1065 (18.5)	0.020 (0.011-0.055)	62.3 (50.0-86.0)
> High School	3236 (68.9)	0.011 (0.011-0.029)	67.4 (55.1-88.2)
**Race/Ethnicity**			
Non-Hispanic white	2225 (70.2)	0.011 (0.011-0.030)	65.1 (53.3-87.6)
Non-Hispanic-black	1116 (9.3)	0.032 (0.011-0.090)	70.4 (58.0-88.7)
Mexican-American	668 (7.8)	0.016 (0.011-0.039)	65.2 (53.6-87.5)
Other	1381 (12.6)	0.018 (0.011-0.041)	62.5 (50.4-81.2)
**BMI**			
Under-/ normoweight	1401 (025)	0.011 (0.011-0.027)	59.9 (51.3-75.3)
Overweight	1836 (36.1)	0.015 (0.011-0.036)	70.0 (55.1-91.1)
Obese	2153 (38.9)	0.018 (0.011-0.047)	67.3 (53.5-89.2)
**Physical activity (METs-hour/week) ^†^**			
1^st^ tertile	2074 (34.7)	0.017 (0.011-0.039)	62.6 (50.3-82.1)
2^nd^ tertile	1739 (33.5)	0.011 (0.011-0.032)	65.8 (53.2-88.5)
3^rd^ tertile	1577 (31.8)	0.015 (0.011-0.039)	69.1 (56.6-90.0)
**Smoking**			
Never	3730 (68.5)	0.011 (0.011-0.032)	64.6 (53.2-86.4)
Ex-smoker	1660 (31.6)	0.018 (0.011-0.047)	67.9 (53.6-86.4)
**Alcohol consumption**			
Never	890 (12.8)	0.011 (0.011-0.035)	56.4 (46.1-70.4)
exdrinker	1220 (20.4)	0.017 (0.011-0.044)	65.7 (53.6-85.4)
Current	2238 (51.8)	0.015 (0.011-0.033)	71.7 (56.7-91.9)
Unknow	1042 (15.0)	0.015 (0.011-0.038)	59.7 (49.3-75.2)
**Diet quality**			
Excellent/very Good	1837 (37.4)	0.011 (0.011-0.028)	63.7 (53.1-83.2)
Good	2322 (42.2)	0.016 (0.011-0.370)	66.2 (53.1-88.0)
Fair/poor	1231 (20.4)	0.019(0.011-0.056)	69.3 (54.7-90.7)
**Diagnosed disease**			
Cardiovascular disease	607 (9.5)	0.018 (0.011-0.049)	59.7 (44.9-78.8)
Respiratory disease	856 (16.4)	0.015 (0.011-0.042)	61.1 (51.3-78.7)
Musculoskeletal disease	1592 (29.0)	0.016 (0.011-0.039)	56.6 (46.7-74.4)
Cancer	597 (13)	0.011 (0.011-0.026)	60.1 (48.2-82.3)
Hypertesion	2347 (39.6)	0.017 (0.011-0.039)	62.3 (49.1-83.6)
Diabetes	994 (14.2)	0.018 (0.011-0.043)	60.9 (49.1-82.5)
**Number of drug treatments**			
None	1987 (37.2)	0.015 (0.011-0.036)	65.6 (53.3-87.8)
1-2	687 (13.4)	0.015 (0.011-0.030)	63.9 (53.6-86.4)
>2	2716 (49.4)	0.016 (0.011-0.037)	65.9 (53.3-85.6)

^**†**^ Cutoff values (men/women) were: ≤12/≤4 (tertile 1), 12 to ≤48 /4.1 to ≤28 (tertile 2), and >48/28 (tertile 3) METs-hour/week.

**Figure 1:**
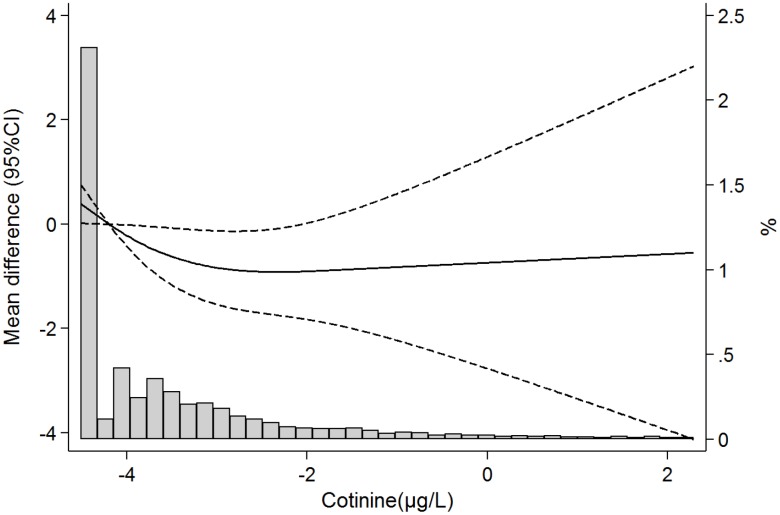
**Mean differences (95%confidence intervals) in grip strength according to serum cotinine concentrations (ng/mL) based on restricted cubic splines with knots at the 10^th^, 50^th^, and 90^th^ percentile of serum cotinine distribution.** The reference value is set at the 25^th^ percentile of the cadmium distribution. Models are adjusted for age, sex, race/ethnicity, education, place of birth, tobacco smoke, alcohol consumption, diet quality, physical activity, BMI, cardiovascular disease, respiratory disease, musculoskeletal disease, cancer, hypertension, diabetes and number of drug treatments. Lines represent predicted values (thick line) and 95% confidence intervals (dashed lines), and vertical bars represent the histogram of log-transformed serum cotinine distribution.

In models adjusted for sociodemographic, lifestyle and clinical risk factors (Model C), and comparing participants in the highest vs the lowest quartile of serum cotinine, combined grip strength decreased by 1.41 kg (-2.58, -0.24); p-trend across quartiles=0.02 (Table 3). Figure 1 shows the dose-response association of serum cotinine with grip strength. As observed, strength declines were observed even at very low levels of exposure, and there were no major departures from linearity (p-values for the linear and non-linear components were 0.04 and 0.11, respectively).

**Table 3:** Beta coefficient (95% confidence interval) for grip strength in kg, by serum cotinine concentration.

	**Serum cotinine concentration quartiles (ng/mL)**				
	**Q1 (≤0.011)**	**Q2 (0.015-0.02)**	**Q3 (0.021-0.047)**	**Q4 (0.048-9.9)**	**p-trend**
**No.**	2275	627	1187	1278	
**Model A**	1.00	0.26 (-1.13, 1.65)	-0.56 (-1.79, 0.67)	-1.09 (-2.24, 0.07)	0.0
**Model B**	1.00	0.14 (-1.25, 1.53)	-0.77 (-2.03, 0.49)	**-1.32** (-2.44, -0.20)	**0.02**
**Model C**	1.00	0.18 (-1.16, 1.52)	-0.86 (-2.19, 0.46)	**-1.41** (-2.58, -0.24)	**0.02**

Model A adjusted for age (continuous), sex, race-ethnicity (Non-Hispanic white, Non-Hispanic black, Mexican-American, Other), educational level (<high school, high school, > high school), and place of birth (US, other).

Model B further adjusted for tobacco smoke (never, ex-smoker), alcohol consumption (never, ex–drinker, current, unknown), diet quality (excellent/very good, good, fair/poor) and physical activity (sex-specific tertiles).

Model C further adjusted for BMI, cardiovascular disease, respiratory disease, musculoskeletal disease, cancer, hypertension, diabetes and number of drug treatments.

**Figure 2:**
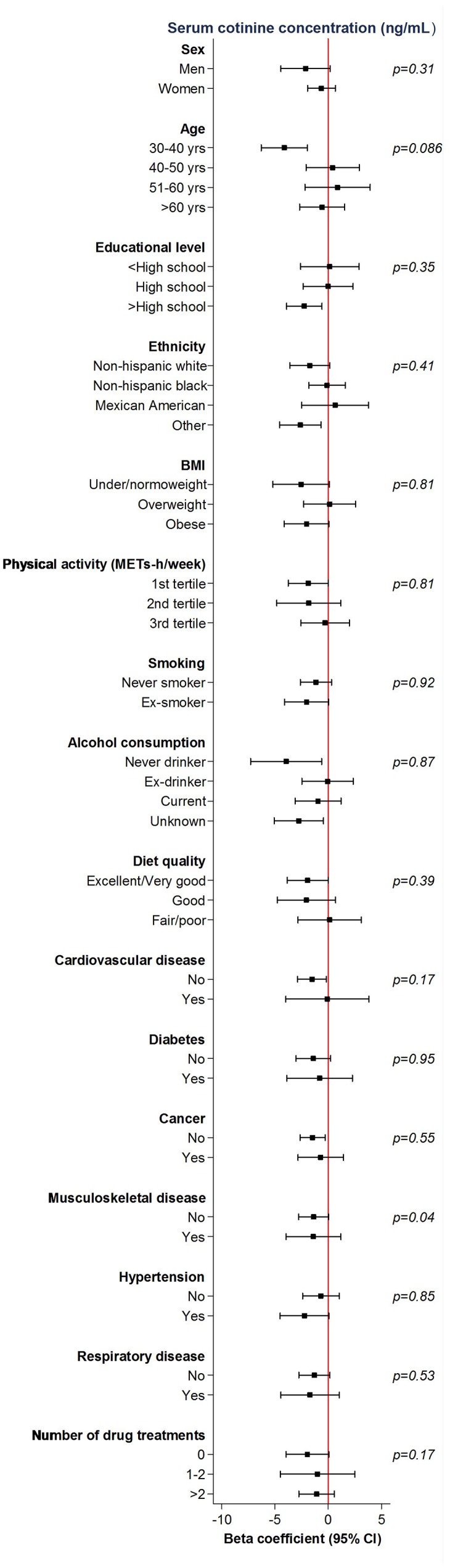
**Mean differences (95%confidence intervals) in grip strength comparing the fourth to the first quartile of cotinine concentrations (ng/mL) by participant’s characteristics.** Models are adjusted for age, sex, race/ethnicity, educational level, place of birth, tobacco smoke, alcohol consumption, diet quality, physical activity, BMI, cardiovascular disease, respiratory disease, musculoskeletal disease, cancer, hypertension, diabetes and number of drug treatments.

Figure 2 shows results for the highest vs the lowest quartile of serum cotinine across participantʼs characteristics. Although we observed no interactions with age, sex, education, lifestyle factors or BMI, we did observe some effect modification by musculoskeletal disease. In particular, the association was only maintained among individuals without musculoskeletal disease: results for the second, third and highest versus the lowest quartile of cotinine were: -0.58 kg (-2.39- 1.23); -1.89 kg (-3.44, -0.34) and -1.37 kg (-2.80, 0.05); p-trend=0.01.

Lastly, additional adjustment for serum albumin, total protein intake, serum testosterone, and glomerular filtration rate in participants with this information available did not modify substantially our main findings (Supplementary Table 1).

## DISCUSSION

In this sample of middle-aged and older non-smokers adults of the U.S. population, exposure to SHS was associated with a decrease in grip strength.

There is in vitro evidence that cigarette smoke induces skeletal muscle damage through atrophy of oxidative muscle fibers, impaired synthesis of muscle proteins, and over-expression of atrophy related genes [21–23]. Studies in vivo have also observed increased muscular oxidative stress and systemic inflammation in mice exposed to cigarette smoke [24, 25]. A comparison of skeletal muscle properties and fatigue resistance between 40 smokers and 45 non-smokers found out that, although muscle mass and contractile properties were similar in smokers and non-smokers, smokers suffered from greater peripheral muscle fatigue (measured as torque decline during a series of repetitive contractions) than non-smokers [26]. Also a longitudinal study with 963 men and women aged >30 years from the *Mini-Finland health examination survey*, who were followed for up to 22 years, showed that former smoking at baseline and persistent tobacco use during follow-up were associated with accelerated grip strength declines [12]. In the same line, a meta-analysis of 12 cross-sectional and case-control studies with a total of 22515 participants showed an association between self-reported active smoking and sarcopenia [27]. Despite this evidence, there is only one recent publication evaluating the potential association between SHS exposure and handgrip strength [19]; in this publication, based on the *English Longitudinal Study of Aging*, the authors found an inverse association between saliva cotinine concentrations and performance on several indicators of physical capacity (including declined grip strength) in older adults. Our study supports the association between SHS and declined grip strength while extends this observation to middle-aged adults (30-60 years). Moreover, it suggests that there is no apparent risk-free level of exposure to SHS.

Of interest is that the dose-response association between SHS exposure and grip strength was only observed among individuals with no arthritis or osteoarthritis. Although these are posthoc findings, and so they need to be interpreted with caution, they may reflect the fact that among individuals with musculoskeletal disease there are other more deterministic factors (i.e. pain, physical inactivity) modifying muscle strength.

Strengths of this study include the national representativeness of the sample and the high quality study protocol and laboratory methods, and the inclusion not only of old but also young adults. To ensure that only nonsmokers were included in the analyses, we applied strict criteria that excluded participants who acknowledged active smoking or whose cotinine levels were indicative of tobacco use, or who had missing responses to the smoking questions or cotinine concentrations. Additionally, our results were consistent in various sensitivity analyses.

Among the limitations of our study are the cross-sectional design and the potential for residual confounding, despite models were adjusted for many relevant variables. More importantly, a single measurement of cotinine only reflects SHS exposure over the previous 1-2 days, so it is an imperfect surrogate of long-term exposure; there is recent evidence for epigenetic biomarkers of long-term cumulative exposure, but unfortunately these are not available in NHANES [28]. Finally, due to the relatively small proportion of participants heavily exposed to SHS (with concentrations close to 10ng/mL), results are hard to interpret at these concentrations.

In the US adult population aged 30 years an over, combined grip strength declined around 1.42 kg every 2.5 years, which is similar to the observed decline in strength comparing participants in the highest vs the lowest quartile of serum cotinine. Although the clinical relevance of such a decline at the individual level is uncertain, at the population level small changes in the distribution of muscle strength, resulting from the widespread exposure to SHS, may have an important impact in functional disability. Future prospective studies should evaluate the link between SHS exposure and functional disability.

## MATERIALS AND METHODS

### Study population

NHANES is a program of the National Center for Health Statistics designed to assess the health and nutritional status of adults and children in the United States [29]. The present analysis used data from 8621 adults aged > 30 years who participated in NHANES 2011-2014 from whom around 90% (7723) had information about grip strength. To ensure that the sample included non-smokers only, we excluded 352 participants with missing serum cotinine data, and 1801 current smokers (reporting having smoked at least 100 cigarettes in their entire life and were active smokers at the time of the interview; or who had serum cotinine levels ≥10 ng/mL). We also excluded 180 participants with missing data on potential confounders, leaving 5390 participants for analyses.

The study was approved by the National Center for Health Statistics Research Ethics Review Board, and written informed consent was obtained from all participants.

### Study variables

### Grip strength

Grip strength was measured by trained personnel using a Takei Dynamometer Model T.K.K.5401 (Takei Scientific Instruments Co., Niigata, Japan). Each hand was tested three times, alternating hands with a 60-second rest between measurements. Combined grip strength was calculated by NHANES as the sum of the largest reading from each hand expressed in kg.

### Tobacco smoke exposure (serum cotinine concentration)

Serum cotinine was measured using high performance liquid chromatography/atmospheric pressure chemical ionization tandem mass spectrometry. The limit of detection (LOD) was 0.015ng/mL, and values under this limit were divided by the square root of 2. Serum cotinine levels were log-transformed due to their skewed distribution, and categorized into quartiles.

### Other variables

We used information from a number of self-reported variables including age, sex, education (< high school, high school, > high school), race/ethnicity (Non-Hispanic White, Non-Hispanic Black, Mexican-American, Other), physical activity (sex-specific tertiles of METs-hour/week), smoking (never smoker, ex-smoker), alcohol consumption (never, ex-drinker, current drinker, unknown), diet quality (based on the question “in general, how healthy is your overall diet?”), number of drug treatments (none, ≤2 and >2), and history of physician-diagnosed chronic conditions: cardiovascular disease (coronary heart disease, heart failure, heart attack, angina, stroke), respiratory disease (asthma, chronic bronchitis, emphysema), musculoskeletal disease (arthritis, osteoarthritis) and cancer. Hypertension was defined as a self-reported physician diagnosis, current use of anti-hypertensive medication, or a clinical blood pressure reading 140/90 mmHg. Likewise, type 2 diabetes mellitus was based as a self-reported physician diagnosis, current use of anti-diabetic medication, or fasting glucose ≥126 mg/dL. Glomerular filtration rate (GFR) was estimated using the Chronic Kidney Disease Epidemiology Collaboration (CKD-EPI) equation that incorporates subjects´ measures of serum creatinine, age, race and sex. Weight and height were measured in standardized conditions, and the body mass index (BMI) calculated dividing weight in kg by squared height in meters. Subjects were then classified as “underweight/ normoweight” (BMI<25 kg/m^2^), “overweight” (BMI ≥25 to <30 kg/m^2^) or “obese” (IMC≥ 30 kg/m^2^).

### Statistical analysis

We performed all statistical analyses in Stata version 13.0, by using the survey command to account for the complex sampling design and weights in NHANES.

We first used frequency distributions to describe the distribution of participant characteristics by serum cotinine quartiles. Then, we depicted the distribution of serum cotinine and combined grip strength for participants, overall and by their main sociodemographic, lifestyle and clinical factors. To evaluate the association of serum cotinine with grip strength, we estimated the mean difference (95%confidence interval (CI)) in strength by quartiles of serum cotinine concentrations using linear regression models. Tests for trend were performed for ordinal serum quartiles in regression models using integer values (0 to 3). Next, to evaluate potential departures from linearity, we ran restricted cubic spline models with knots at the 10^th^ (0.011 ng/mL), 50^th^ (0.018 ng/mL), and 90^th^ (0.0184 ng/mL) percentiles of cotinine distribution in all participants.

We built three models with progressive levels of adjustment: Model A adjusted for the main sociodemographic variables (age, sex, race/ethnicity, education, and place of birth); Model B further adjusted for lifestyle factors (physical activity, tobacco smoke, alcohol consumption, and self-reported diet quality); and Model C additionally adjusted for BMI, morbidity (cardiovascular disease, musculoskeletal disease, respiratory disease, cancer, hypertension, diabetes) and number of drug treatments.

We evaluated the consistency of our findings across categories of potential confounders using interaction models, and differences across strata were tested with interaction terms. Additionally, to reduce potential residual confounding, we performed analyses with further adjustment for nutrition factors (serum albumin and total protein intake), as these have been associated with both exposure to SHS and decreased grip strength [10, 11]. Likewise, because both passive smoking and grip strength have been associated with kidney function, we adjusted our models for glomerular filtration rate [30, 31]. Finally, since there is some evidence suggesting that cotinine may inhibit testosterone breakdown [32], and low testosterone levels are associated with decreased muscle strength [33, 34], we run models that additionally adjusted for serum testosterone concentrations [32].

## Supplementary Material

Supplementary Table 1
