# Supplementary material for: Exposure to secondhand tobacco smoke is associated with reduced muscle strength in US adults
Source: Aging (Albany NY). 2019 Dec 9;11(24):12674–84. doi: 10.18632/aging.102594 (PMC6949081; doi:10.18632/aging.102594)
Supplement: Supplementary Table 1 [file aging-11-102594-s001..pdf]

SUPPLEMENTARY TABLE

Supplementary Table 1. Sensitivity analyses. Results are beta coefficient (95% confidence interval) for grip strength (kg), by serum cotinine concentrations.

		Serum cotinine concentration quartiles (ng/mL)				
	**n**	**Q1 ≤0.011**	**Q2 0.015-0.02**	**Q3 0.021-0.047**	**Q4 0.048-9.9**	**p- trend**
**SA [1]**	5,357	1.00	0.19 (-1.13, 1.51)	-0.74 (-2.07, 0.59)	**-1.14 (-2.27, -0.00)**	**0.05**
**SA [2]**	5,080	1.00	0.33 (-1.07, 1.74)	-0.94 (-2.34, -0.46)	**-1.37 (-2.59, -0.15)**	**0.02**
**SA [3]**	5,324	1.00	0.23 (-1.12, 1.58)	-0.87 (-2.17, 0.44)	**-1.35 (-2.57, -0.12)**	**0.03**
**SA [4]**	5,357	1.00	0.23 (-1.10, 1.56)	-0.84 (-2.13, 0.46)	**-1.27 (-2.44, -0.09)**	**0.03**

Analyses are adjusted for the same variables as Model C in table 3 plus: SA[1]- Serum albumin; SA[2]- Total protein intake; SA[3]- Serum testosterone; and SA[4]- Glomerular filtration rate
